# Intratumoral heterogeneity drives acquired therapy resistance in a patient with metastatic prostate cancer

**DOI:** 10.1038/s41698-024-00773-w

**Published:** 2024-12-02

**Authors:** Dena P. Rhinehart, Jiaying Lai, David E. Sanin, Varsha Vakkala, Adrianna Mendes, Christopher Bailey, Emmanuel S. Antonarakis, Channing J. Paller, Xiaojun Wu, Tamara L. Lotan, Rachel Karchin, Laura A. Sena

**Affiliations:** 1 The Sidney Kimmel Comprehensive Cancer Center at Johns Hopkins University, Baltimore, MD USA; 2 Institute for Computational Medicine at Johns Hopkins University, Baltimore, MD USA; 3 University of Minnesota Masonic Cancer Center, Minneapolis, MN USA

**Keywords:** Prostate cancer, Tumour heterogeneity, Cancer therapeutic resistance

## Abstract

Metastatic prostate cancer (PCa) is not curable due to its ability to acquire therapy resistance. Theoretically, acquired therapy resistance can be driven by changes to previously sensitive cancer cells or their environment and/or by outgrowth of a subpopulation of cancer cells with primary resistance. Direct demonstration of the latter mechanism in patients with PCa is lacking. Here we present a case report as proof-of-principle that outgrowth of a subpopulation of cancer cells lacking the genomic target and present prior to therapy initiation can drive acquired resistance to targeted therapy and threaten survival in patients with PCa.

## Introduction

Cancer is described to possess acquired therapy resistance when it grows despite treatment and despite prior growth restriction by that treatment. Acquired therapy resistance renders most systemic cancer therapies useful for a limited duration of time and as such is a major driver of death from cancer. Fundamentally, acquired therapy resistance can be driven by changes to previously sensitive cancer cells or their environment (e.g., acquisition of *AR* amplification or activating mutations following therapies that inhibit the androgen receptor, AR) and/or by the outgrowth of cancer cells with primary resistance (i.e., clonal selection)^1,2^. Tumor heterogeneity is a prerequisite for acquired therapy resistance by clonal selection. It has long been appreciated that PCa has a high degree of heterogeneity, as approximately 75% of primary PCa are multifocal with distinct foci often containing unique genomic features^3–6^. Recently it was shown that distinct PCa clones derived from the primary tumor can produce independent metastases, even prior to the selective pressure of therapy, suggesting that PCa metastases can propagate heterogeneity derived from the primary^6^. Intratumoral heterogeneity (ITH) enabling clonal selection and acquired therapy resistance has been demonstrated in patients with medulloblastoma^7^, but to our knowledge has not previously been demonstrated in patients with PCa.

Currently two classes of genomic alterations can be targeted therapeutically in PCa, including those leading to DNA mismatch repair deficiency (MMRd) or hypermutation by anti-PD1, and those leading to DNA homologous recombination deficiency by PARP inhibition. We previously reported that MMRd prostate cancer (PCa) has a high biochemical response rate (approximately 65%), but low durability of response (median progression-free survival of approximately 6 months) to anti-PD1 immunotherapy^8^. High ITH of neoantigens has been shown to drive resistance to anti-PD1 in a mouse model of MMRd cancer^9^ and was associated with non-response to anti-PD1 in patients with lung cancer^10^. Mechanisms governing acquired therapy resistance to anti-PD1 in patients with MMRd PCa are unknown. Here we describe a patient case that demonstrates ITH with clonal selection can be a mechanism of acquired immunotherapy resistance in a patient with MMRd PCa.

## Results

### Clinical course of the case patient

**Fig. 1:**
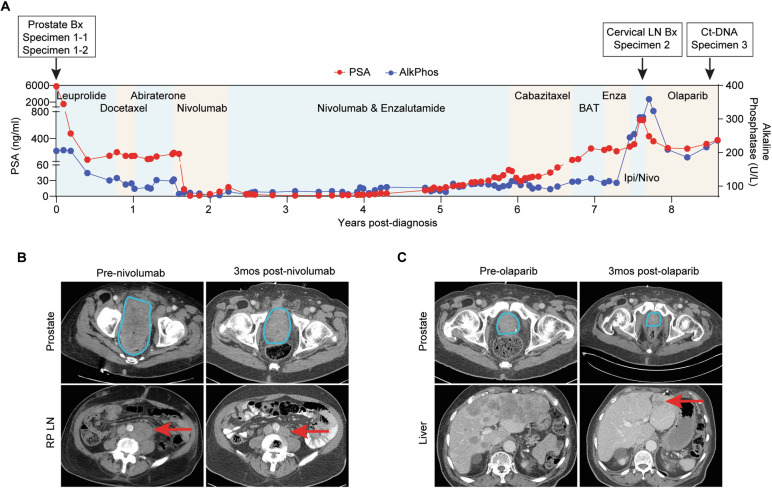
Clinical course of the case patient. **A** Trend of prostate-specific antigen (PSA) and alkaline phosphatase (AlkPhos) over time in the case patient. Timing and duration of exposure to systemic therapies are indicated. BAT bipolar androgen therapy, Enza enzalutamide, Ipi/Nivo ipilimumab and nivolumab. **B** Serial axial computed tomography (CT) directly prior to and following 3 months of treatment with nivolumab. RP LN retroperitoneal lymph node. **C** Serial axial CT directly prior to and following 3 months of treatment with olaparib. Blue line indicates border of prostate/prostate tumor and red arrow indicates tumor in (**B**, **C**).

**Fig. 2:**
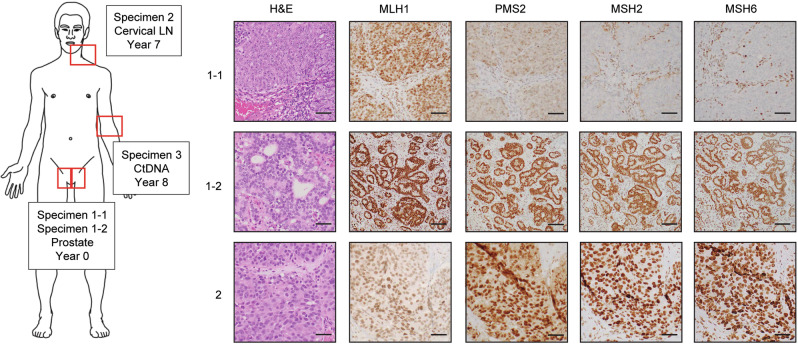
Heterogeneity of mismatch repair protein expression within and between tumors. Hematoxylin and eosin (H&E) stain and immunohistochemistry for mismatch repair proteins in specimens obtained at indicated locations and times post-diagnosis. Cartoon adapted from “Silhouette humain asexue anterieur posterieur” available without copyright on Wikimedia Commons.

A 76-year-old man presented with gross hematuria. Evaluation revealed prostate-specific antigen (PSA) elevation to 5786 ng/ml and widely metastatic cancer to the axial and appendicular skeleton, and mediastinal, iliac, and periaortic lymph nodes with a large prostate mass with maximal dimension 14 cm. Transurethral resection of prostate (Specimen 1) revealed high-grade prostate adenocarcinoma (Gleason 5 + 4 = 9) with genomic deletion of *MSH2* by next generation sequencing (NGS) and loss of MSH2 and its binding partner MSH6 protein expression by immunohistochemistry (IHC), consistent with MMRd PCa. Germline NGS was negative for a pathogenic germline mutation in the Invitae 84-gene panel. The patient’s PSA steeply declined to a plateau of 150 ng/ml after initiation of androgen deprivation therapy with leuprolide, following which he was treated with docetaxel and subsequently abiraterone without a PSA response to either agent (Fig. 1A). Unfortunately, the patient continued to experience significant hematuria and consequent urinary obstruction, requiring suprapubic catheter placement and blood transfusions. The patient then began treatment with the anti-PD1 agent nivolumab through the NCI-MATCH trial for patients with MMRd cancer. This resulted in a 99.4% decline in his PSA over 2 months from 172 ng/ml to 1 ng/ml (Fig. 1A), marked reduction in tumor volume (Fig. 1B), and resolution of hematuria. However, his cancer rapidly acquired resistance over the next 4 months with a steadily rising PSA. Enzalutamide was then added to nivolumab treatment, which resulted in a 93% decline in the PSA with sustained response over several years. Following progression on nivolumab and enzalutamide, he was treated with cabazitaxel, bipolar androgen therapy^11^, a second trial of enzalutamide, and nivolumab in combination with ipilimumab, without notable response to any agent. The patient had become cachectic, weak, and unable to engage in most activities. Restaging scans revealed increasing bone metastases, lymphadenopathy, and development of numerous liver metastases (Fig. 1C). At this point, >7 years following diagnosis, a biopsy of an enlarged cervical lymph node was obtained (Specimen 2). Pathological analysis surprisingly revealed prostate adenocarcinoma with retained expression of MMR proteins (Fig. 2; Specimen 2). NGS using the FoundationOne CDx panel showed a tumor mutational burden (TMB) of 10 muts/Mb, microsatellite stable status, *BRCA2* deletion, *CBL* mutation, *TMPRSS2-ERG* fusion, *MYC* gain, and *RAD21* gain, and no alteration of *MSH2*. Given *BRCA2* loss, the patient was treated with the PARP inhibitor olaparib, which resulted in a 63% decline in his PSA over 6 months from 677 ng/ml to a nadir of 251 ng/ml (Fig. 1A), near-resolution of his liver metastases (Fig. 1C), healthy weight gain, and restoration of his physical function. Unfortunately, the PSA subsequently began rising and restaging scans showed progression of cancer in the lymph nodes, bones, and liver. NGS of circulating tumor DNA (ctDNA; Specimen 3) using the FoundationOne Liquid CDx panel showed a ctDNA tumor fraction of 5.1% with estimated TMB 8 muts/Mb, microsatellite stable status, *TMPRSS2-ERG* fusion, pathogenic mutations in *AR, CHEK2, NF1, PIK3CA*, and *TP53*, and no alterations to *MSH2* nor *BRCA2*. Notably, this test does not measure copy number alterations of *MSH2* and its median limit of detection of *BRCA2* copy number loss is a tumor fraction of 30.4%, according to the FDA-filed technical report.

### Heterogeneity of mismatch repair protein expression within and between tumors

Given discordance in *MSH2* between the primary tumor (Specimen 1) and the lymph node metastasis (Specimen 2), stored tissue from the primary tumor specimen was re-examined. Written informed consent was received prior to study. On re-examination of the primary, two distinct regions of tumor were observed. The first region (Specimen 1-1) exhibited high-grade adenocarcinoma (Gleason 5 + 4 = 9) with loss of MSH2 protein and its binding partner MSH6 (Fig. 2). The second newly identified region of the primary tumor (Specimen 1-2) exhibited more differentiated adenocarcinoma (Gleason 4 + 3 = 7) with retained expression of MMR proteins, similar to the lymph node metastasis (Specimen 2) obtained 7 years later (Fig. 2).

### Late metastatic disease is derived from a lower grade MMRp area of the primary tumor

**Fig. 3:**
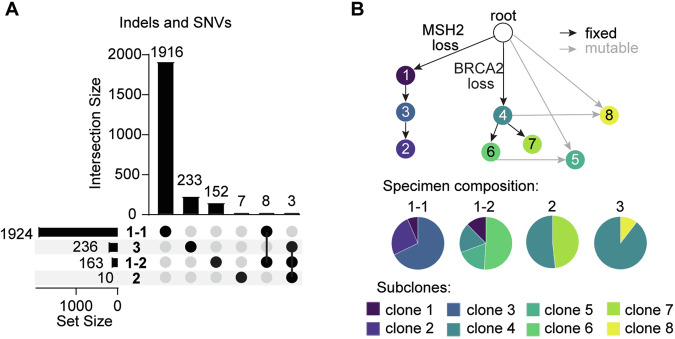
Late metastatic disease is derived from a lower grade MMRp area of the primary tumor. **A** UpSet plot depicting unique and shared indels and single nucleotide variants (SNVs) across specimens. The total number of detected mutations in each specimen (Set Size) are shown on the left. Number of unique (dots) and shared (dots connected by lines) mutations (Intersection Size) are indicated in the bar graph. **B** Composite tumor evolution analysis using PICTograph across specimens. Specimen composition derived from tree 1.

To enable study of the clonal relationships between the 4 specimens, whole exome sequencing was performed on Specimens 1-1 and 1-2 and normal tissue. Remarkably, while Specimen 1-1 was identified to have >1900 genomic alterations due to its MMRd status, none of these were shared with Specimen 2 or Specimen 3 (Fig. 3A). In contrast, Specimen 1-2 shared multiple alterations with Specimens 2 and 3 and exhibited *BRCA2* deletion similar to Specimen 2 (Fig. 3A). Tumor evolution analysis using PICTograph (see methods) identified 8 cancer subclones across these 4 specimens. Four trees describing the ancestral relationships between these subclones were identified, each with equal probability (Supplementary Fig. 1). All trees depict an early branching event separating ancestry of clones with *MSH2* loss from those with *BRCA2* loss (Fig. 3B). Notably, Specimen 1-1 was comprised of subclones 1–3, none of which were identified in Specimen 2 or Specimen 3 (Fig. 3B). In contrast, Specimen 1-2 was comprised of subclones 1 and 4–6, with subclone 4 shared with Specimen 2 and Specimen 3 (Fig. 3B). This suggests that this patient’s metastatic PCa >7 years following diagnosis was derived from a low-grade MMR-proficient region of the primary tumor that was present at diagnosis and was not clonally related to the high-grade MMR-deficient region of the primary tumor.

## Discussion

This case is proof-of-concept that ITH can drive acquired resistance to anti-PD1 therapy in a patient with MMRd PCa. This patient possessed two distinct PCa clones at diagnosis, one with *MSH2* loss and one with *BRCA2* loss. Serendipitously, these alterations represent the two classes of currently targetable genomic alterations in PCa. Therefore, response to these targeted therapies enabled us to assess the extent to which each clone was dominant across metastatic sites. It seems that the clone with *MSH2* loss dominated at the time of initiation of anti-PD1, given the >99% decline in his PSA and radiographic and clinical response with this therapy. However, the patient’s cancer subsequently acquired resistance with rising PSA and subsequent biopsy of a metastatic lymph node showed outgrowth of the clone with *BRCA2* loss. It seems that, at this time, the clone with *BRCA2* loss dominated, given the >60% decline in his PSA and radiographic and clinical response with PARP inhibition, including near-resolution of liver metastases. This demonstrates that the clonal dominance of PCa can shift over time due to selective pressure of therapy, driving acquired therapy resistance. These targeted therapies appeared to be highly effective in this patient, as the targeted genomic alterations were not detected at relapse and may have been eliminated, although technical limitations of testing preclude high confidence in this. Nonetheless these treatments were insufficient to cure owing to the presence and outgrowth of untargeted clones.

Notably, both clones that gave rise to metastases were present in the primary tumor at the time of diagnosis. Thus, the value of repeat genomic evaluation following progression on anti-PD1 was to identify a driver mutation that was previously missed in sequencing of the primary tumor. Therefore, while this case demonstrates utility of repeat genomic evaluation following development of therapy resistance, perhaps a more proactive approach is more comprehensive genomic evaluation of the primary tumor at diagnosis, as recently suggested by Warner et al.^6^. A limitation of comprehensive genomic evaluation of the primary tumor is that this may lead to uncertainty of which clone or genomic driver is dominant and most urgently life-threatening to which therapy should be directed. Serial circulating tumor DNA (ctDNA) sequencing is a very promising clinical tool, as ctDNA may represent DNA from different tumor sites and cancer clones within those sites to both identify driver mutations and estimate the abundance of these driver mutations to infer clonal dominance.

Of note, this case is reminiscent of a case previously reported by Haffner et al. who identified a lethal PCa clone to be derived from a low-grade area of the primary tumor^12^. Similarly, the dominant and life-threatening clone after >7 years of therapy in this patient seemed to be derived from a low-grade area of the primary tumor. A caveat to this conclusion is that the primary tumor was not completely evaluated given that only TURP samples (rather than a radical prostatectomy sample) were obtained. Therefore, it is possible that unsampled areas of the primary tumor contained cancer of higher grade clonally related to the MMR-proficient BRCA2-deficient clone. Nonetheless, this may suggest that cancers with genomic features that can drive therapy resistance can threaten the patient’s survival, even despite less aggressive histological features at diagnosis.

The broader clinical significance of this case is not known, as it is a single case report that may represent a rare scenario. We previously reported that loss of MSH2 appears homogenous by IHC of the dominant nodule of primary PCa^13^, but this does not preclude the possibility of heterogeneity across nodules. We also previously reported a case of MMRd PCa metastatic to the brain parenchyma in which acquired resistance to anti-PD1 was clearly not driven by outgrowth of an unrelated subclone^14^. Future studies should address how frequently ITH (as well as intertumoral heterogeneity) drives acquired resistance to targeted therapies in patients with PCa. Given that prostate cancer is known to have a high degree of genomic heterogeneity, one might predict that adoption of a “precision medicine” approach to treatment of metastatic prostate cancer in which targeted monotherapies are utilized would enable this type of acquired therapy resistance to become common. A related question is whether acquired therapy resistance enabled by tumor heterogeneity of PCa can be prevented. Answers would necessitate tools to accurately measure heterogeneity and clonal dominance within and across tumor sites, as well as strategies to reduce heterogeneity. One can speculate that the survival benefit of first-line combination therapy strategies in metastatic PCa, including combination systemic and combination systemic and focal therapies^15^, may be due to reduction of cancer clonal diversity and limitation of future outgrowth of resistance clones.

In summary, this case demonstrates that tumor heterogeneity can drive acquired resistance to targeted treatment of PCa by enabling outgrowth of an untargeted clone and has implications for future study of therapeutic strategies to eliminate metastatic PCa.

## Methods

### Mismatch repair protein immunohistochemistry and interpretation

We performed the MMR protein IHC staining using the Ventana Benchmark Ultra using Ventana MMR IHC Panel antibodies according to the manufacturer’s instructions (Roche/Ventana Medical Systems, Tucson, AZ). The specimens were screened for loss of MSH6 (catalog #790-5092, clone: SP93, Rabbit Monoclonal Primary Antibody), PMS2 (catalog #790–5094, clone: A16-4, Mouse Monoclonal Primary Antibody), MSH2 (catalog # 790–5093, clone: G219–1129, Mouse Monoclonal Primary Antibody), and MLH-1 (catalog # 790–5091, clone: M1, Mouse Monoclonal Primary Antibody). We considered a specimen as having MMR protein loss if there was diffuse loss of staining in tumor nuclei, with intact nuclear staining in tumor stromal and immune cells as an internal control.

### Whole exome sequencing (WES) and analysis

DNA was isolated from regions of interest of the primary tumor and thyroid for germline evaluation using the AllPrep® DNA/RNA FFPE Kit (Qiagen, Cat. No. 80234) and quantified using the Qubit™ dsDNA HS Assay Kit (Invitrogen, REF Q32854). WES was performed by Admera Health with 2 × 150 estimated 26 million paired end reads in each direction per sample. BAM files for lymph node and circulating tumor DNA samples sequenced at Foundation Medicine (CDx panel) were obtained and FASTQ files extracted. Samples were demultiplexed, quality checked, duplicates marked, filtered and aligned to genome build GRCh38 using pre-established pipelines implemented via snakePipes^16^, with bwa v_0.7.17, deeptools v_3.3.2, seqtk v_1.3, pigz v_2.3.4, snpsplit v_0.3.4, samtools v_1.10, fastqc v_0.11.9, cutadapt v_2.8, trim-galore v_0.6.5, multiqc v_1.8, fastp v_0.20.0, umi_tools v_1.0.1 and qualimap v_2.2.2d. Somatic mutations were called with the matched normal sample using the gatk toolkit (v4.4.0.0) following the somatic short variant discovery best practices workflows^17^. Variant annotation was done using OpenCRAVAT (v2.5.0) and variants of the following types were selected: frameshift insertion, frameshift deletion, inframe deletion, inframe insertion, missense variant, splice site variant, stop gain, and stop loss^18^. Mutations with a variant read count of at least 7 and a total read depth of at least 15 in at least one sample were included for downstream analysis. The mutations reported in the FoundationOne Liquid CDx panel were recovered and added to analysis through visual inspection using IGV^19^. Somatic copy number alterations were called using FACETS (v0.6.2)^20^. Tumor subclonal reconstruction was performed using PICTograph (v1.3.0)^21^. Four trees of equal probability were identified and shown in Supplementary Fig. 1. A composite tree summarizing these 4 trees is shown in the main figure.

### Study approval

We have complied with all relevant ethical regulations including the Declaration of Helsinki. This study was approved by the institutional review board at Johns Hopkins University. Informed consent was obtained from the patient prior to publication of this report.

## Supplementary information

Supplementary Material

## Data Availability

The data and materials used in the current study are available from the corresponding author upon reasonable request. The raw sequencing data are not publicly available due to informed consent restrictions.

## Code availability

The tumor evolution modeling package used in this study is open source and is available here: https://github.com/KarchinLab/pictograph.
