# Supplementary material for: Intratumoral heterogeneity drives acquired therapy resistance in a patient with metastatic prostate cancer
Source: NPJ Precis Oncol. 2024 Dec 2;8:275. doi: 10.1038/s41698-024-00773-w (PMC11612198; doi:10.1038/s41698-024-00773-w)
Supplement: Supplementary file 1 — Supplementary Material [file 41698_2024_773_MOESM1_ESM.pdf]

Supplementary Material

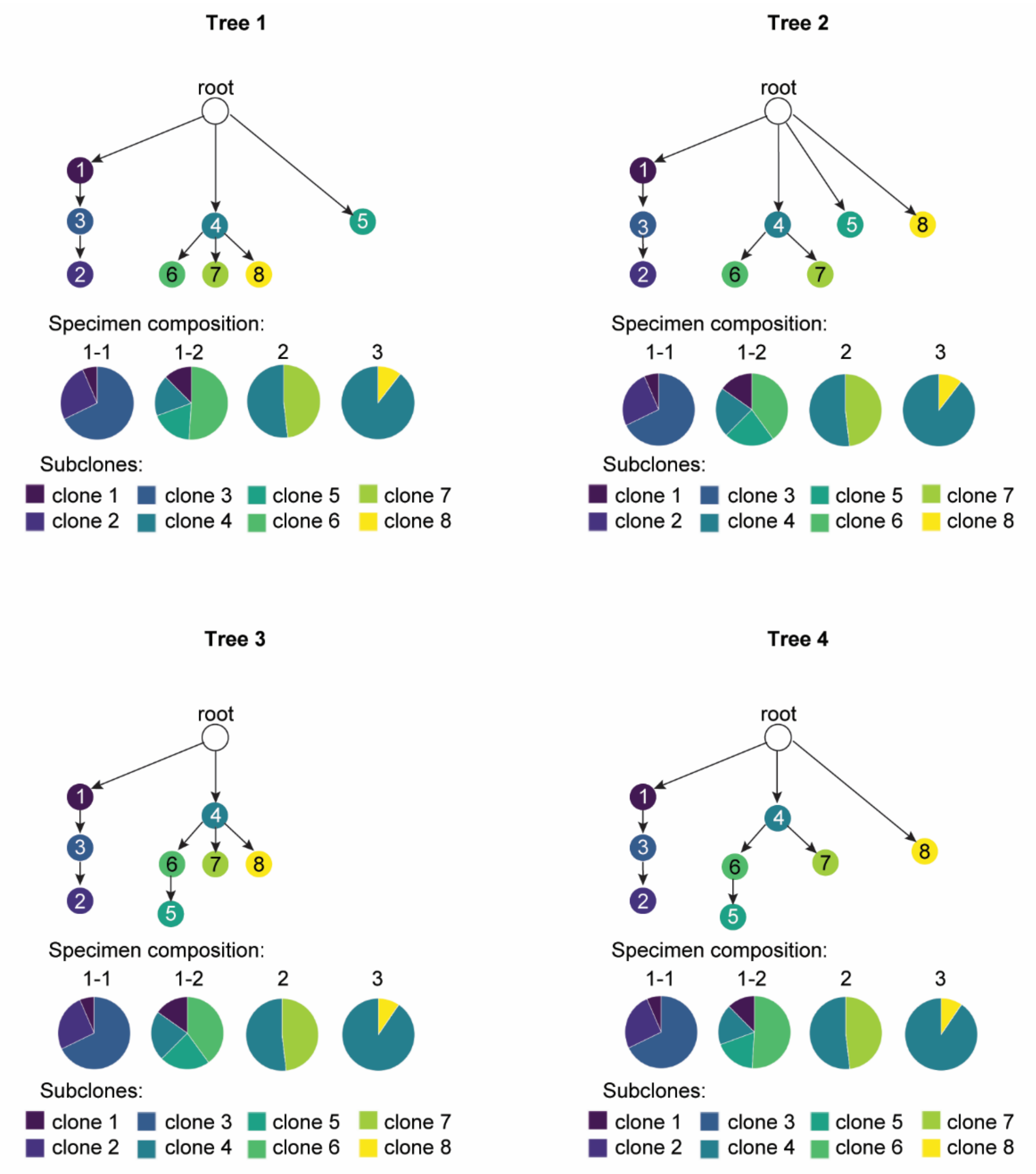

Supplementary Figure 1. Subclonal relationships between samples, related to Fig 3B. Tumor subclonal reconstruction using PICTograph identified 4 trees of equal probability. Specimen composition derived from each tree is shown.
